# Phytochemical and Bioactivity Studies on *Hedera helix* L. (Ivy) Flower Pollen and Ivy Bee Pollen

**DOI:** 10.3390/antiox12071394

**Published:** 2023-07-06

**Authors:** Nisa Beril Sen, Etil Guzelmeric, Irena Vovk, Vesna Glavnik, Hasan Kırmızıbekmez, Erdem Yesilada

**Affiliations:** 1 Department of Pharmacognosy, Faculty of Pharmacy, Yeditepe University, Kayisdagi Cad., Atasehir, Istanbul 34755, Türkiye; 2 Laboratory for Food Chemistry, National Institute of Chemistry, Hajdrihova 19, SI-1000 Ljubljana, Slovenia

**Keywords:** *Hedera helix* L., ivy, bee pollen, chemical profiling, antioxidant activity, xanthine oxidase inhibitory activity, HPTLC-bioautography

## Abstract

Bee pollen, known as a ‘life-giving dust’, is a product of honeybees using flower pollen grains and combining them with their saliva secretions. Thus, flower pollen could be an indicator of the bee pollen botanical source. Identification of bee pollen sources is a highly crucial process for the evaluation of its health benefits, as chemical composition is directly related to its pharmacological activity. In this study, the chemical profiles, contents of phenolic marker compounds and pharmacological activities of *Hedera helix* L. (ivy) bee pollen samples from Türkiye and Slovenia, as well as ivy flower pollen grains, were compared. High-performance thin-layer chromatography (HPTLC) analyses revealed that pollen samples, regardless of where they were collected, have similar chemical profiles due to the fact that they have the same botanical origins. Marker compounds afzelin, platanoside and quercetin-3-*O*-β-glucopyranosyl-(1→2)-β-galactopyranoside, common to both bee pollen and flower pollen, were isolated from bee pollen, and their structures were elucidated by nuclear magnetic resonance (NMR) and mass spectrometry (MS). These three compounds, as well as chlorogenic acid and 3,5-dicaffeoylquinic acid (found in flower pollen), were quantified using high-performance liquid chromatography (HPLC) analyses. *In vitro* tests and effect-directed analyses were used to evaluate the xanthine oxidase inhibition and antioxidant activity of the marker compounds and extracts from flower pollen and bee pollen. This is the first report comparing chemical profiles and related bioactivities of the flower pollen and bee pollen of the same botanical origin, as well as the first report of the chemical profile and related bioactivities of ivy flower pollen.

## 1. Introduction

*Hedera helix* L. (ivy, Araliaceae) is an evergreen perennial plant that blooms from September to November in the northern hemisphere and is naturally grown or cultivated around the world. Its leaves are rich in saponins (e.g., hederacoside C) and phenolic compounds (chlorogenic acid, 3,5-caffeoylquinic acid, 3,4-caffeoylquinic acid, rutin, hyperoside, etc.), and they have a medical importance [1]. The pharmaceutical products containing standardized extract of ivy leaves are commonly sold over-the-counter as an expectorant for relieving respiratory tract infections [2]. Apart from the research on leaves, there is limited scientific research on the chemical composition and related bioactivity of other plant parts. There are only two reports on phenolic compounds in ivy flowers [3,4]. In addition to ivy flowers, phenolic compounds were also investigated in ivy fruits [3,4] and also in ivy leaves [4]. Chlorogenic acid [4], ferulic acid [3,4], *p*-coumaric acid [3], isoquercitrin [4], rutin (rutoside) [3,4], quercetin (quercetol) [3,4], and kaempferol [3] were found in flowers [3,4] and fruits [3,4], while quercitrin [3] was found only in flowers [3]. One of these reports [4] provides data about antimicrobial and antioxidant activities of extracts from leaves, flowers and fruits. Since ivy blooms in late season, it provides pollen as a valuable food source for honeybees before the winter season [5]. In spite of this importance, there is no information about the chemical profile and related bioactivity of the ivy pollen.

Bee pollen has been known as the ‘only perfectly complete food’ because it contains essential amino acids required by the human. Besides, it meets all the requirements of dietetic recommendations—and a person can live healthy by eating only bee pollen [6]. Bee pollen contains phenolic compounds, mainly phenolic acids and flavonoids, which originate from flowers [7]. Thus, it should be emphasized that the chemical composition of bee pollen is directly related to its botanical source, which influences the bioactivity profile of bee pollen. Therefore, the botanical source of bee pollen should be taken into consideration for the standardization of bee pollen extracts used in apitherapy. However, there are no data comparing chemical profiles and the related bioactivity of flower pollen and bee pollen of the same botanical origin.

Identification of pollen grains in samples is commonly performed by palynological (microscopic) analysis, which is limited by: (1) the necessity of having an expert in pollen analysis; (2) the necessity of consulting a comprehensive reference pollen atlas; (3) the great similarity of the pollen grains, even when they belong to different plant genus [8]. Due to these three drawbacks pollen analysis is not applicable in most of the laboratories. Therefore, there is a need for other identification methodologies (e.g., methods based on chromatographic techniques) as an alternative to palynological analysis. High-performance thin-layer chromatography (HPTLC) is one of the most widely applied techniques for the chemical profiling of natural compounds in plants [9,10,11,12,13,14,15,16] and plant-related materials [14,17,18,19]. Compared to other chromatographic techniques HPTLC has several advantages that enable: analyses of several samples simultaneously under the same conditions; subsequent detection at different wavelengths in absorption and fluorescence mode; use of a variety of post-chromatographic derivatizations; evaluation of fingerprints on the whole R_F_ scale (from the start (application position) to the front). HPTLC is also the only chromatographic technique which provides the chromatogram in the form of an image, which can further be processed by image analysis for quantification [9,10,11,14,20]. HPTLC-image analysis in combination with chemometric methods is applicable for the grouping of samples containing specific compounds based on their botanical origin, as was demonstrated in a study of phenolic compounds in bee pollen samples [18]. Additionally, various bioactivity analyses (effect-directed analyses/detection) can be performed directly on the HPTLC plates after the separation or dot-spot analyses. Effect-directed analyses on HPTLC plates include: (1) chemical (antioxidant activity) and enzyme-based assays (e.g., acetylcholinesterase, glucosidase, amylase, xanthine oxidase, tyrosinase) [21]; (2) cell-based assays (antimicrobial, antipathogenic, genotoxic, hormonal (estrogenic, androgenic, both agonistic and antagonistic activity) [22]. Such effect-directed analyses are very efficient and can even be applied for analyses of crude extracts.

Xanthine oxidase (XO, EC 1.17.3.2) is an enzyme responsible for uric acid formation. XO catalyzes the oxidation of hypoxanthine to xanthine and xanthine to uric acid. If uric acid crystals are overproduced, the inflammatory response can be triggered, resulting in joint arthritis known as gout. XO is also responsible for generating oxygen-derived free radicals that may result in an imbalance between pro-oxidants and antioxidants, causing some people to be more prone to diseases such as cancer [23]. Anti-gout drugs (e.g., allopurinol), acting as inhibitors of XO activity, may have some side effects such as hepatitis, nephropathy and allergic reactions. Therefore, researchers are more focused on combined therapies with natural compounds to minimize the side effects [24]. Phenolic compounds in natural products act not only as antioxidants but also as XO inhibitors.

Related to the facts mentioned above, the aims of this study were: (1) chemical profiling of ivy flower pollen and bee pollen of the same botanical origin (samples from Türkiye and Slovenia); (2) isolation by column chromatography and structural elucidation (one-dimensional (1D) and two-dimensional (2D) nuclear magnetic resonance (NMR) spectroscopy and mass spectrometry (MS)) of the marker compounds from bee pollen; (3) development and validation of the high-performance liquid chromatography (HPLC) method for the quantification of the marker compounds in flower pollen and bee pollen; (4) determination of the antioxidant activity and inhibition of xanthine oxidase (XO) by the extracts from flower pollen and bee pollen; (5) HPTLC-bioautographic analyses and evaluation of antioxidant activity and inhibition of XO by the marker compounds, as well as the extracts from flower pollen and bee pollen.

## 2. Materials and Methods

### 2.1. Chemicals

All chemicals used were at least of analytical grade. Methanol (HPLC grade and analytical grade), ethanol, acetonitrile (HPLC grade), ethyl acetate, dichloromethane, glacial acetic acid, formic acid (98–100%), 2,2′-azino-bis(3-ethylbenzothiazoline-6-sulfonic acid) diammonium salt (ABTS), 2,2-diphenyl-1-picrylhydrazyl (DPPH·), 2,4,6-tri(2-pyridyl)-s-triazine (TPTZ) and xanthine oxidase (XO) were acquired from Sigma-Aldrich (Steinheim, Germany). Polyethylene glycol 400 (PEG 400), *o*-phosphoric acid (85%), sodium acetate, di-sodium hydrogen phosphate heptahydrate and copper (II) sulfate pentahydrate were obtained from Merck (Darmstadt, Germany). Ethylenediaminetetraacetic acid (EDTA), iron (III) chloride and ammonium acetate were from Fluka (Steinheim, Germany), while 2-aminoethyl diphenylborinate (NP) was from Alfa Aesar (Karlsruhe, Germany). Hydrochloric acid and sodium phosphate monobasic dihydrate were purchased from Riedel-de Haen (Seelze, Germany), while nitro blue tetrazolium chloride (NBT) was from Cayman Chemical Company (Ann Arbor, Michigan, USA). Standards of 3,5-dicaffeoylquinic acid, chlorogenic acid (≥95%) and trolox ((±)-6-hydroxy-2,5,7,8-tetramethylchromane-2-carboxylic acid) (97%) were acquired from Sigma-Aldrich (Steinheim, Germany), while allopurinol (98%) was purchased from Acros Organics (United Kingdom). Neocuproine was acquired from Sigma-Aldrich, and xanthine (99%) was acquired from Alfa Aesar (Kandel, Germany). Distilled water was used for extractions and all analyses except for HPLC analyses for which ultrapure water obtained from Simplicity UV purification system (Millipore, Darmstadt, Germany) was used.

### 2.2. Samples

Ivy (*H. helix*) flowers were collected in the forest in Bayramiç (Türkiye), and androecia, containing anthers composed of pollen sacs, were separated from the flowers. Bee pollen samples were obtained from the professional beekeepers who placed their beehives in Ordu (Türkiye; BP-TR) and Hrastnik (Slovenia; BP-SI) districts. Palynological analysis was applied according to the standard methodology [25]. Investigated bee pollen samples were classified according to Barth [26] as dominant pollen (>45%), secondary pollen (15–45%), important minor pollen (3–15%) and minor pollen (<3%). Based on this classification ivy pollen was found in the bee pollen samples from Ordu and Hrastnik in the proportion of 88.7% and 92.3%, respectively. All samples were kept at −20 °C.

### 2.3. Preparation of Standard Solutions

All standard solutions were prepared in methanol. Stock solution of standards (chlorogenic acid and 3,5-dicaffeoylquinic acid) and isolated compounds (quercetin-3-*O*-β-glucopyranosyl-(1→2)-β-galactopyranoside, afzelin, and platanoside) were prepared at a concentration of 200 μg/mL. Equal volumes (500 µL) of stock solutions of all five compounds were mixed to obtain a standard mixture (MIX) for HPTLC analyses.

Additional stock solutions were prepared for HPLC analyses: 250 μg/mL for quercetin-3-*O*-β-glucopyranosyl-(1→2)-β-galactopyranoside, chlorogenic acid and platanoside; 500 μg/mL for afzelin and 3,5-dicaffeoylquinic acid. These stock solutions were further diluted with methanol to prepare seven working solutions for each standard in the following concentration ranges: 2.5–250 μg/mL for quercetin-3-*O*-β-glucopyranosyl-(1→2)-β-galactopyranoside, chlorogenic acid and platanoside; 5–500 μg/mL for afzelin and 3,5-dicaffeoylquinic acid. Then, these seven working standard solutions were mixed together (from the lowest to highest concentration) to prepare standard mixtures for the calibration curves (0.5–50 μg/mL for the first three and 1–100 μg/mL for the last two compounds).

### 2.4. Preparation of Sample Test Solutions

Bee pollen and flower pollen samples (5 g) were extracted with 80% ethanol_(aq)_ (50 mL) using ultrasound-assisted extraction (30 min). The solutions obtained was filtered by using filter paper and concentrated under reduced pressure at 40 °C to obtain hydroalcoholic extracts. Hydroalcoholic extracts (100 mg) were dissolved in methanol (5 mL) using a sonicator and filtered through 0.45 μm hydrophilic-regenerated cellulose (RC) membrane filters (Minisart, Sartorius Stedim Biotech, Goettingen, Germany) to obtain sample test solutions (STSs, 20 mg/mL). Undiluted STSs were used for HPTLC analyses, while SSTs diluted with methanol (DSTSs) were used for HPLC analyses (0.25–2 mg/mL) and for bioactivity analyses (1–2.5 mg/mL for DPPH• assay, ferric reducing antioxidant power (FRAP) assay, cupric reducing antioxidant capacity (CUPRAC) assay and ABTS assay; 0.01–1 mg/mL for superoxide radical scavenging (SOD) activity and xanthine oxidase (XO) inhibitory activity assays).

The following assignments were used to distinguish the STSs and DSTSs of the bee pollen samples based on the collection country (Türkiye—TR and Slovenia—SI): STS-TR, DSTS-TR, STS-SI and DSTS-SI. Assignments used for the STS and DSTS of flower pollen were: STS-FP and DSTS-FP.

### 2.5. Isolation and Structure Elucidation of the Marker Compounds from the Bee Pollen Sample

**Figure 1:**
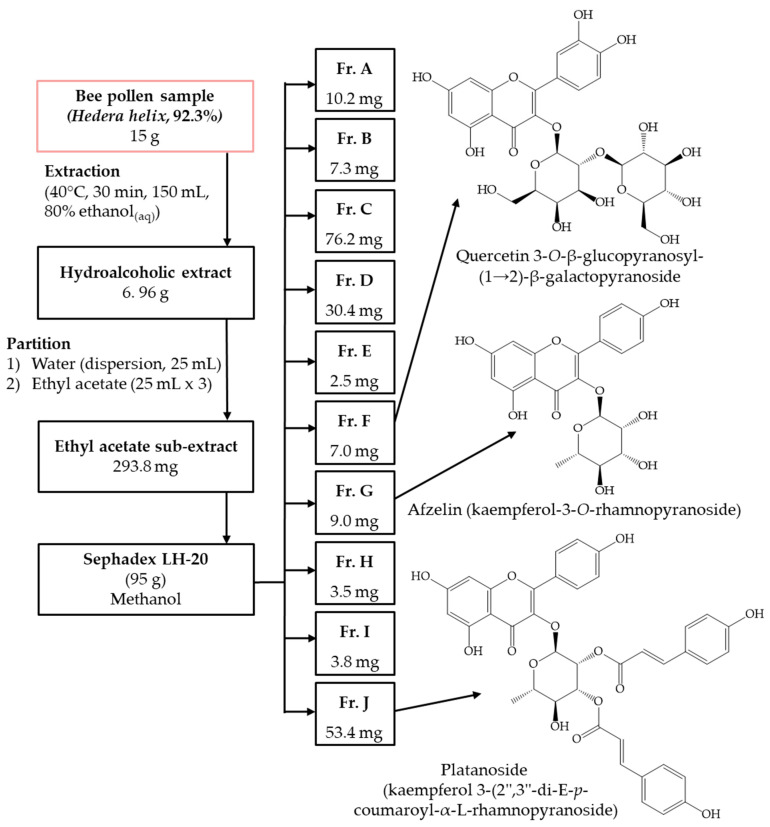
The isolation scheme with chemical structures of compounds isolated (fractions (Fr.) F, G and J).

The bee pollen sample from Slovenia (15 g) was extracted with 80% ethanol_(aq)_ (150 mL) for 30 min in an ultrasonic bath at 40 °C. After filtration, the solvent was evaporated under vacuum at 40 °C to yield crude hydroalcoholic extract (6.96 g). Then, the hydroalcoholic extract was suspended in distilled water (25 mL) and partitioned with ethyl acetate (25 mL × 3). The ethyl acetate fraction (293.8 mg) was applied onto Sephadex LH-20 (95 g) chromatographic column with methanol to afford three compounds (Figure 1). The structures of these compounds were elucidated by NMR (1D and 2D) and MS.

### 2.6. MS/MS Analyses

The isolated quercetin-3-*O*-β-glucopyranosyl-(1→2)-β-galactopyranoside, afzelin and platanoside (1 mg) were first dissolved in methanol (1 mL), and the solutions obtained (1 mg/mL) were further diluted with methanol. Working solutions obtained (0.02 mg/mL) were analyzed by means of an LTQ Velos mass spectrometer with dual-pressure linear ion trap mass analyzer (Thermo Fisher Scientific, Waltham, MA, USA) using a heated electrospray ionization source (HESI) in negative ion mode to ionize the compounds. MS parameters were as follows: flow rate 10 µL/min, heater temperature 200 °C, capillary temperature 350 °C, sheath gas 60 a.u. (arbitrary units), auxiliary gas 10 a.u., sweep gas 0 a.u., spray voltage 2.5 kV, S-Lens RF level 69% and capillary voltage 38.8 V [16]. MS spectra were obtained in the range of 100–2000 *m/z*. The fragmentation of parent ions was performed with 35% collision energy and the isolation width of 1.0 *m/z*. Collected data were evaluated using Xcalibur software (version 2.1.0, Thermo).

### 2.7. HPTLC Analyses

MIX and SSTs (20 mg/mL, 2 µL and 5 µL) of bee pollen samples (STS-TR and STS-SI) and flower pollen sample were applied on 20 cm × 10 cm glass backed HPTLC silica gel 60 F_254_ plates (Merck, Art. No. 1.05642) with a semi-automatic applicator Linomat 5 (Camag, Muttenz, Switzerland) equipped with a 100 µL Hamilton syringe. Applications were performed as 8 mm bands (15.4 mm apart), 8 mm from the bottom of the plate and 15 mm from the left edge. The plate was developed up to 7 cm in a saturated (20 min) twin-trough chamber (20 cm × 10 cm, Camag) with a developing solvent, ethyl acetate–dichloromethane–acetic acid–formic acid–water (100:25:10:10:11, *v/v/v/v/v*) [27]. After drying the plate in a stream of cold air, the plate was heated on a TLC plate heater (Camag) at 100 °C for 3 min and immersed into NP derivatization reagent and after drying also into PEG 400 derivatization reagent [28] by a Chromatogram Immersion Device III (Camag) for 3 s. Documentation of the plate images was performed using a Visualiser (Camag) after development (at 254 nm and 366 nm), after derivatization with NP reagent (at 366 nm) and after enhancement of the fluorescence by PEG 400 reagent (at 366 nm). The winCATS program was used to operate all the instruments (Camag, Version 128 1.4.8.2031).

### 2.8. HPLC Analyses

HPLC analysis was performed using the 1260 Infinity HPLC system (Agilent, Waldbronn, Germany), consisting of a quaternary pump, an autosampler, a thermostatted column compartment and a diode array detector (DAD). The HPLC system was operated by ChemStation software. HPLC analysis was carried out on a Zorbax RP18 Column (4.6 mm × 250 mm I.D., 5 μm particle size, Agilent). The column temperature was set to 25 °C. Mobile phase A [*o*-phosphoric acid-water (0.1:99.9, *v*/*v*)] and mobile phase B (acetonitrile) were degassed and filtered before analyses. The following gradient elution was applied: 15–18% B (0–5 min), 18–41% B (5–15 min), 41–55% B (15–25 min), 55–80% B (25–27 min), 80% B (27–29 min), and 80–15% B (29–31 min). The flow rate was 1 mL/min. The injection volume was 10 μL. Three different acquisition wavelengths were used for quantitative analyses: (1) 260 nm for quercetin-3-*O*-β-glucopyranosyl-(1→2)-β-galactopyranoside and afzelin; (2) 310 nm for platanoside; (3) 330 nm for chlorogenic acid and 3,5-dicaffeoylquinic acid. This newly developed HPLC method was validated according to the International Conference on Harmonisation (ICH) 1995 guidelines [29]. The validated method was then applied for the quantification of all five compounds investigated in DSTSs.

### 2.9. In Vitro Bioactivity Analyses

Bioactivity analyses (antioxidant, superoxide radical scavenging, and xanthine oxidase inhibitory activity) were performed for: (1) DSTSs of bee pollen samples; (2) DSTS flower pollen; (3) isolated compounds (afzelin, platanoside and quercetin-3-*O*-β-glucopyranosyl-(1→2)-β-galactopyranoside); (4) chlorogenic acid and 3,5-dicaffeoylquinic acid. The same range of concentration (1–2.5 mg/mL) of DSTSs was applied for DPPH, FRAP, CUPRAC and ABTS assays. For isolated compounds, chlorogenic acid and 3,5-dicaffeoylquinic acid, the concentration ranges were as follows: 0.1–0.2 mg/mL for DPPH; 0.05–0.2 mg/mL FRAP and ABTS; 0.025–0.2 mg/mL for CUPRAC; 0.01–1 mg/mL for superoxide radical scavenging and XO inhibitory activity assays. The antioxidant activities were expressed as mg of trolox equivalents (TE) per g of hydroalcoholic extract (TE/g hydroalcoholic extract). Half-maximal inhibitory concentration values (IC_50_) were calculated for superoxide radical scavenging and XO inhibitory activity. Inhibition values (%) were calculated based on absorbances (A) measured against blank solutions by using the following formula:

Inhibition (%): (1 − (A_Sample_ − A_Sample Blank_)/(A_Control_ − A_Control Blank_)) × 100. (1)

Details about blanks, controls (substrate and enzyme solutions) and control blank (substrate solution) are described in the following subsections.

#### 2.9.1. DPPH• Assay

Equal volumes (20 μL) of the DSTSs, trolox standard solutions (3.125–100 μg/mL) and methanol (as a blank) were placed in separate wells of a 96-well microplate, which was followed by the addition of DPPH solution (0.1 mM, 280 μL) to each well. After incubation in the dark at room temperature for 30 min, the absorbance was measured at 530 nm [30].

#### 2.9.2. FRAP Assay

FRAP solution was freshly prepared by mixing 1 volume of iron (III) chloride solution (2 × 10^−2^ M), 1 volume of TPTZ solution (1 × 10^−2^ M) and 10 volumes of sodium acetate buffer (pH 3.6) solution. FRAP solution (280 µL) and the DSTSs (20 µL) or trolox standard solutions (20 µL; 3.125–100 μg/mL) or water as a blank (20 µL) were added in separate wells of a 96-well microplate, and after 6 min the absorbance was measured at 593 nm [31].

#### 2.9.3. CUPRAC Assay

The CUPRAC method [32] was applied. Copper (II) sulfate pentahydrate (10 mM, 85 µL), neocuproine (7.5 mM, 85 µL) ammonium acetate buffer solution (85 µL, pH 7) and water (51 µL) were inserted in each well of a 96-well microplate. Then, either 43 µL of the DSTSs or trolox standard solutions (3.125–200 μg/mL) or water as a blank were added into each well. After incubation at room temperature for 30 min, the absorbance was measured at 450 nm.

#### 2.9.4. ABTS Assay

ABTS assay [33] was slightly modified and then applied. Equal volumes (20 µL) of the DSTSs, trolox standard solutions (6.25–100 μg/mL) or methanol (as a blank) were placed in separate wells of a 96-well microplate plate, and ABTS reagent (280 µL) was added. The absorbance was measured at 734 nm, after incubating the well plate for 6 min at room temperature.

#### 2.9.5. Xanthine Oxidase Inhibitory Activity

XO inhibitory activity was carried out as described in Ref. [34] with minor changes. Sodium phosphate buffer (50 mM, pH 7.5, 75 µL) was placed in the wells of a 96-well microplate; this was followed by the DSTSs in different concentrations (25 µL); followed by freshly prepared XO solution (0.2 U/mL in phosphate buffer solution, 25 µL); followed by water (25 µL). Incubation of the reaction mixture at 37 °C for 15 min was followed by the addition of a substrate solution (0.15 mM xanthine, 50 µL) into the mixture and incubation at 37 °C for 30 min until the reaction was terminated by the addition of hydrochloric acid (0.5 M, 50 µL). Finally, the absorbance was measured at 290 nm against a blank, containing all reagents, except the enzyme solution. Allopurinol at different concentrations in the range from 15 to 200 µg/mL was used as a positive control.

#### 2.9.6. Superoxide Radical Scavenging (SOD) Activity

SOD activity was performed as described in Ref. [27]. DSTSs (20 µL) and allopurinol (10–200 µg/mL; used as the positive control) were added in separate wells of a 96-well microplate; this was followed by the substrate solution (mixture of 0.4 mM xanthine and 0.24 mM NBT, 80 µL); followed by XO solution (50 mU/mL in sodium phosphate buffer (pH 7.5), 80 µL). After incubation at 37 °C for 20 min the reaction was terminated by hydrochloric acid (0.6 M, 80 µL) and absorption was measured at 560 nm against a blank solution containing all reagents except XO.

### 2.10. HPTLC-Effect-Directed Analyses (EDA)

#### 2.10.1. HPTLC-DPPH•

The HPTLC plates developed as described in Section 2.7. HPTLC analyses were dipped into DPPH solution (0.1%) for 3 s using a Chromatogram Immersion Device III (Camag). The plates were left to dry in the air in the dark and were documented under white light after 30 min. The compounds having antioxidant activity appeared as yellow bands on the purple background.

The image of the HPTLC plate captured at white light illumination after HPTLC-DPPH analyses was for image analyses converted to a different format using WinCATS software (Camag) and then converted to videodensitograms in fluorescence mode using VideoScan TLC/HPTLC Evaluation Software (Version 1.02.00) (Camag).

#### 2.10.2. HPTLC-Xanthine Oxidase Inhibitory Activity

The XO bioautography assay was carried out as described in Ref. [35]. The HPTLC plate developed as described in Section 2.7. HPTLC analyses was immersed into a derivatization chamber with a mixture of phosphate buffer solution (pH 7.6) containing EDTA (1 mM), NBT (1 mM) and XO (0.1 U/mL), and incubated at 37 °C for 30 min in the dark. After drying in the air, the HPTLC plate was dipped into a phosphate buffer solution containing xanthine (1.5 mM) and incubated at 37 °C for 30 min in the dark. After drying in the air, the HPTLC plate was documented at white light in RT mode immediately (0 min), as well as at 5, 10, 15, 20, 30, 45, 60, 90, and 120 min, subsequently. The compounds having XO inhibitory activity were detected as white/yellow zones on a purple background. Allopurinol (8 µg on the plate) was used as the positive control.

### 2.11. Statistical Analyses

Each assay for bioactivity and quantitative analysis was repeated thrice. The average values and standard deviations (SD) were calculated by using Microsoft Excel 2013, and the results were expressed as average value ±SD. The analytical data obtained from tests were evaluated by using one-way analysis of variance (ANOVA). Tukey’s test was applied to appraise the differences (*p* < 0.05) by Minitab 17.

## 3. Results and Discussion

Chemical profiling of the STS-TR and STS-SI prepared from ivy bee pollen samples collected in Türkiye and Slovenia was first performed on the HPTLC silica gel F_254_ plates using NP detection reagent followed by PEG detection reagent. The most intense bands obtained at the same R_F_ values in both STS-TR and STS-SI presenting unidentified marker compounds were selected for isolation and evaluation of their structures.

### 3.1. Isolation and Structural Elucidation of Compounds Isolated from BP-SI

The ethyl acetate soluble fraction of the crude hydroalcoholic extract prepared from the bee pollen was subjected to gel filtration chromatography on Sephadex LH-20 to obtain unidentified marker compounds. The exact chemical structures of the isolates (Figure 1) were elucidated as quercetin 3-*O*-β-glucopyranosyl-(1→2)-β-galactopyranoside [36], afzelin (kaempferol-3-*O*-rhamnopyranoside) [37], and platanoside (kaempferol 3-(2″,3″-di-E-*p*-coumaroyl-α-L-rhamnopyranoside) [38] by comparing their NMR (one-dimensional (1D) and two-dimensional (2D)) and MS (Table 1) data with those published in the literature. These identified compounds were found for the first time in bee pollen samples that originated from ivy.

Direct infusion MS and MS^n^ analyses of compounds isolated (Table 1) showed characteristic base ions and fragment ions for compounds isolated. Fragmentation of quercetin-3-*O*-β-glucopyranosyl-(1→2)-β-galactopyranoside base ion at *m/z* 626 [M-H]^-^ (Table 1) resulted in ion at *m/z* 301 assigned as quercetin aglycone [39], as well as ions at *m/z* 445 [M-H-180]^-^, 505 [M-H-120]^-^ and 463 [M-H-162]^-^ [40], which corresponded to quercetin with a glucopyranosyl or a galactopyranosyl moiety. Fragmentation of platanoside base ion at *m/z* 723 [M-H]^-^ (Table 1) resulted in the following ions: at *m/z* 285 [M-438-H]^-^ for kaempferol aglycone [39]; at *m/z* 437 [M-286-H]^-^ corresponding to a coumaroyl-rhamnose moiety; at *m/z* 577 [M-146-H]^-^, corresponding to kaempferol with coumaroyl or kaempferol with a rhamnosyl moiety [38,40]. Fragmentation of afzelin base ion at *m/z* 431 [M-H]^-^ (Table 1) resulted in the following ions: at *m/z* 285 [M-H-146]^-^ assigned as kaempferol aglycone; at *m/z* 255 [M-H-176]^-^ corresponding to kaempferol minus CH_2_O^-^ [41]. Additional ions with low relative intensities were detected in the MS spectra of all compounds isolated (Table 1). Because ions at *m/z* 661 (quercetin-3-*O*-β-glucopyranosyl-(1→2)-β-galactopyranoside), *m/z* 759 (platanoside) and *m/z* 467 (afzelin) after fragmentation lost 36 u (Table 1), they were assigned as aglycones with two molecules of water. Such observations were described also for other flavonoids [39,40]. Additionally, dimers of compounds isolated assigned as [2M-H]^-^ were also observed at: *m/z* 1251 (dimer of quercetin-3-*O*-β-glucopyranosyl-(1→2)-β-galactopyranoside); *m/z* 1447 (dimer of platanoside); *m/z* 863 (dimer of afzelin) (Table 1).

### 3.2. HPTLC Chemical Profiling

**Figure 2:**
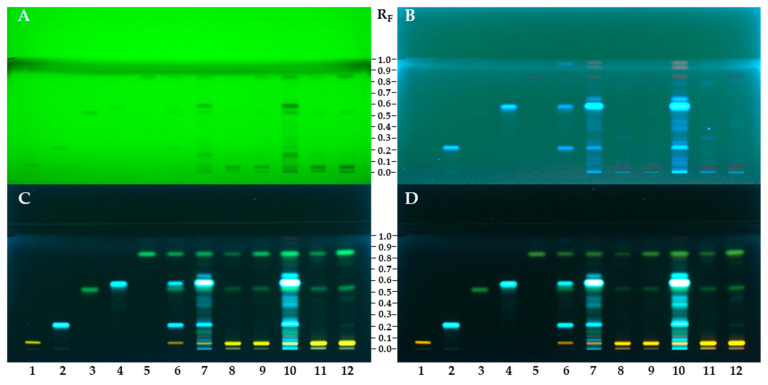
HPTLC silica gel F_254_ plate developed with ethyl acetate–dichloromethane–acetic acid–formic acid–water (100:25:10:10:11, *v/v/v/v/v*), documented before (at 254 nm (**A**) and 366 nm (**B**–**D**)) and after derivatization with NP detection reagent (**C**), followed by enhancement and stabilization of the zones with PEG 400 (**D**). Applications: track 1: quercetin-3-*O*-β-glucopyranosyl-(1→2)-β-galactopyranoside (0.4 µg); track 2: chlorogenic acid (0.4 µg); track 3: afzelin (0.4 µg); track 4: 3,5-dicaffeoylquinic acid (0.4 µg); track 5: platanoside (0.4 µg); track 6: MIX (0.2 µg of each standard); tracks 7 and 10: STS of flower pollen (20 mg/mL; track 7 – 2 µL and track 10 – 5 µL); tracks 8 and 11: STS-TR of bee pollen (20 mg/mL, track 8 – 2 µL and track 11 – 5 µL); tracks 9 and 12: STS-SI of bee pollen (20 mg/mL, track 9 – 2 µL and track 12 – 5 µL).

The chemical fingerprintings of the STS-TR and STS-SI prepared from the bee pollen were comparatively investigated with the STS prepared from the ivy flower, standards and isolated compounds on HPTLC silica gel F_254_ plates before (at 254 nm (Figure 2A) and at 366 nm (Figure 2B)) and after derivatization with NP detection reagent (Figure 2C) followed by enhancement and stabilization of the fluorescence with PEG detection reagent (Figure 2D). As shown in Figure 2D, STS-TR (tracks 8 and 11) and STS-SI (tracks 9 and 12) from bee pollen showed similar profiles with the most intensive chromatographic zones at the R_F_ values of the isolated compounds: quercetin-3-*O*-β-glucopyranosyl-(1→2)-β-galactopyranoside (track 1, orange-colored zone; R_F_ ≈ 0.06), afzelin (track 3, green-colored zone; R_F_ ≈ 0.52) and platanoside (track 5, green-colored zone; R_F_ ≈ 0.84). At the same R_F_ values chromatographic zones with the same color were detected also in the chemical profile of the STS of flower pollen (Figure 2D, tracks 7 and 10), but with higher intensity than in the profiles of the STS-TR and STS-SI. The most intensive chromatographic zones in the STS of flower pollen profile (Figure 2D, tracks 7 and 10) were found at the R_F_ values of chlorogenic acid (Figure 2D, track 2, light blue-colored zone; R_F_ ≈ 0.22) and 3,5-dicaffeoylquinic acid (Figure 2D, track 4, light blue-colored zone; R_F_ ≈ 0.58), which were used as the standards. Although chlorogenic acid and 3,5-dicaffeoylquinic acid were dominant in the flower pollen, they were not detected in bee pollen samples. The qualitative differences between ivy flower pollen and ivy bee pollen chemical profiles may be due to the enzymes present in the bee saliva, resulting in alteration of the compounds. Afzelin, platanoside and quercetin-3-*O*-β-glucopyranosyl-(1→2)-β-galactopyranoside were detected in ivy flower pollen and ivy bee pollen for the first time and can be considered as marker compounds.

Although bee pollen samples were from different countries, their similar chemical profiles indicate that phenolic compounds are directly related with the plant source used by the honeybee. As a consequence, HPTLC chemical profiling in this study may encourage and support future studies focusing on botanical identification of bee pollen samples.

### 3.3. HPLC Analyses

A new HPLC-DAD method was developed for quantification of the compounds investigated (quercetin-3-*O*-β-glucopyranosyl-(1→2)-β-galactopyranoside, chlorogenic acid, platanoside, afzelin and 3,5-dicaffeoylquinic acid) in the ivy bee pollen samples and ivy flower pollen. The method was validated for specificity, linearity, recovery, intraday and interday precision, limit of detection (LOD) and limit of quantification (LOQ).

#### 3.3.1. HPLC Method Validation

##### Specificity

**Figure 3:**
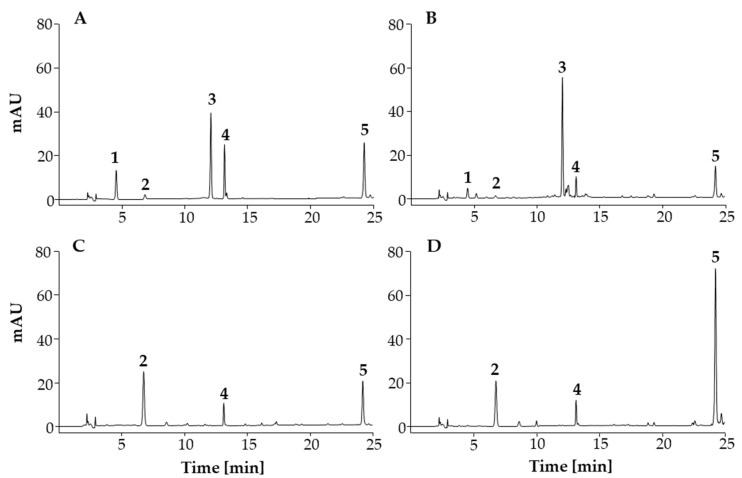
HPLC chromatograms of compounds investigated (**A**), STS of flower pollen (**B**), STS-TR of bee pollen (**C**) and STS-SI of bee pollen (**D**). Peak numbering: chlorogenic acid (**1**), quercetin-3-*O*-β-glucopyranosyl-(1→2)-β-galactopyranoside (**2**), 3,5-dicaffeoylquinic acid (**3**), afzelin (**4**), platanoside (**5**).

The identity of each investigated compound in the samples was verified by comparing its retention time (t_R_) with the t_R_ of the corresponding standard (Figure 3) and overlaying the UV spectrum of each of the compounds investigated with the spectrum of the corresponding standard. The retention times were as follows: 4.6 for chlorogenic acid; 6.8 for quercetin-3-*O*-β-glucopyranosyl-(1→2)-β-galactopyranoside; 12.1 for 3,5-dicaffeoylquinic acid; 13.2 for afzelin; 24.3 for platanoside. The specificity of the method was evaluated by comparison of the chromatogram of each of the compounds investigated with the chromatogram of the blank. Chromatographic peaks for the compounds investigated were not detected in the blank chromatogram, which confirmed the specificity of the method.

##### Linearity of the Calibration Curve, Limit of Detection (LOD), and Limit of Quantification (LOQ)

To establish the calibration curve, seven concentration levels for each standard compound were analyzed in triplicate. The detailed data for the linearity of the calibration curves are presented in Table 2. The obtained correlation coefficient values (r^2^) for the calibration curves are greater than 0.990. LOD and LOQ values (Table 2) were calculated as 3 × (SD/S) and 10 × (SD/S), respectively.

##### Precision

The intraday precision of the HPLC method was evaluated by using a standard mixture solution containing: 5 μg/mL of chlorogenic acid, quercetin-3-*O*-β-glucopyranosyl-(1→2)-β-galactopyranoside and platanoside; 10 μg/mL of 3,5-dicaffeoylquinic acid and afzelin. The mixture was analyzed in three consecutive runs at three different times during the same day. Relative standard deviation (RSD) values are indicated in Table 3. The interday precision was evaluated by repeating the analysis of a standard mixture solution used for examining intraday precision three times on three consecutive days. The intraday precision and interday precision should not exceed 5%. The relative standard deviation values were found in the ranges of 0.019–0.752 for intraday precision and 0.045–0.430 for interday precision (Table 3), indicating that the values found fit the criteria.

##### Accuracy

Recovery of the method at three different levels was calculated in percentages; as a difference between determined and added concentration of the standards in the sample test solution. The concentrations of standards spiked to sample test solutions (BP-TR, BP-SI and FP) are listed in Table 4. The recovery results were between 80.0–106.1% for quercetin-3-*O*-β-glucopyranosyl-(1→2)-β-galactopyranoside, 90.3–103.9% for chlorogenic acid, 72.2–108.4% for 3,5-dicaffeoylquinic acid, 78.4–95.8% for afzelin and 93.1–107.4% for platanoside (Table 4).

#### 3.3.2. Quantitative Analyses

The developed and validated HPLC method was used for the quantitative analyses of the investigated compounds in bee pollen and flower pollen samples. As can be seen in the HPLC chromatograms (Figure 3), all compounds investigated were found in the flower pollen sample (Figure 3B), while chlorogenic acid and 3,5-dicaffeoylquinic acid were not detected in the bee pollen samples (Figure 3C,D). This confirmed the results of the HPTLC analyses (Figure 2). The results of quantitative analyses are presented in Table 5. Quercetin-3-*O*-β-glucopyranosyl-(1→2)-β-galactopyranoside was detected as the dominant compound in the bee pollen samples (≈29 mg/g for BP-TR, ≈24 mg/g for BP-SI), however, this was not the case for the flower pollen sample (Table 5), where much lower concentration (≈4 mg/g) was determined. The dominant compound in the flower pollen sample was 3,5-dicaffeoylquinic acid (≈22 mg/g), which was not detected in the bee pollen samples (Table 5). Both bee pollen samples contained similar concentrations (≈2 mg/g) of afzelin, which concentration was about three times higher in the flower pollen sample (Table 5). In terms of platanoside content a remarkable difference was found between bee pollen samples (≈2 mg/g for BP-TR, ≈7 mg/g for BP-SI).

This is the first report on the content of the phenolic compounds in ivy flower pollen. This report compliments the only two existing reports on the contents of phenolic compounds in ivy flowers [3,4], which include the following phenolic compounds: chlorogenic acid [3], ferulic acid [3], *p*-coumaric acid [3,4], kaempferol, quercetin (quercetol) [3,4], rutin (rutoside) [3,4], quercitrin [3] and isoquercitrin [3].

### 3.4. In Vitro Bioactivity Analyses

#### 3.4.1. Antioxidant Activity Determined by DPPH, FRAP, ABTS, and CUPRAC Assays

DPPH, FRAP, ABTS and CUPRAC assays revealed that phenolic acids (chlorogenic acid and 3,5-dicaffeoylquinic acid) have much higher antioxidant activity than the other compounds investigated (Table 6). Of these four assays the highest antioxidant activity was determined for chlorogenic acid, which was followed by 3,5-dicaffeoylquinic acid and quercetin-3-*O*-β-glucopyranosyl-(1→2)-β-galactopyranoside (Table 6). Afzelin and platanoside had much lower antioxidant activity. As expected, considering the content of phenolic compounds investigated (Table 5), the DSTS of the flower pollen sample had a higher content of investigated phenolic compounds than the DSTSs of the bee pollen samples (Table 5). It also had a higher antioxidant activity than the DSTSs of the bee pollen samples (Table 6). DPPH, FRAP, ABTS and CUPRAC assays showed that the DSTS-SI had higher antioxidant activity than the DSTS-TR (Table 6).

#### 3.4.2. Xanthine Oxidase Inhibitory Activity and Superoxide Radicals Scavenging Activity

Among the compounds investigated, chlorogenic acid, afzelin and 3,5-dicaffeoylquinic acid showed mild-to-moderate XO inhibitory activities, with IC_50_ values ranging from 12.7 to 17.5 µg/mL (Table 7). XO inhibitory activities of chlorogenic acid and 3,5-dicaffeoylquinic acid were also reported by other authors [42,43,44]. Quercetin-3-*O*-β-glucopyranosyl-(1→2)-β-galactopyranoside exhibited low XO inhibitory activity in this study, while platanoside and hydroalcoholic extracts of bee pollen samples had no XO inhibitory activity at the applied concentrations (Table 7). Although both afzelin and platanoside are kaempferol derivatives, afzelin showed XO inhibitory activity, while platanoside showed no activity, which could be due to the bulk groups of coumaric acid esters on platanoside tending to reduce the affinity towards XO.

The enzymatic method was also used to determine SOD activity. Compared to allopurinol as the positive control (IC_50_ 3.0 µg/mL), a significant SOD activity was observed for chlorogenic acid (IC_50_ 1.6 µg/mL) (Table 7). Platanoside showed no SOD activity. The rest of the compounds investigated, as well as hydroalcoholic extracts of the flower and bee pollen samples, showed mild to moderate SOD activity with IC_50_ values ranging from 5 to 63 µg/mL (Table 7).

### 3.5. HPTLC-Effect-Directed Analyses

#### 3.5.1. HPTLC-DPPH Analyses of Antioxidant Activity

**Figure 4:**
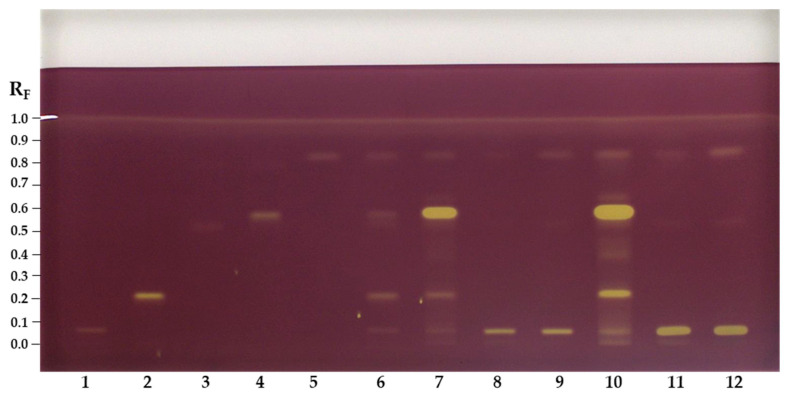
Chromatograms on HPTLC silica gel F_254_ plate developed with ethyl acetate–dichloromethane–acetic acid–formic acid–water (100:25:10:10:11, *v/v/v/v/v*), documented at white light after detection with DPPH reagent. Applications: track 1: quercetin-3-*O*-β-glucopyranosyl-(1→2)-β-galactopyranoside (0.4 µg); track 2: chlorogenic acid (0.4 µg); track 3: afzelin (0.4 µg); track 4: 3,5-dicaffeoylquinic acid (0.4 µg); track 5: platanoside (0.4 µg); track 6: MIX (0.2 µg of each standard); tracks 7 and 10: STS of flower pollen (20 mg/mL; track 7 – 2 µL and track 10 – 5 µL); tracks 8 and 11: STS-TR of bee pollen (20 mg/mL, track 8 – 2 µL and track 11 – 5 µL); tracks 9 and 12: STS - SI of bee pollen (20 mg/mL, track 9 – 2 µL and track 12 – 5 µL).

HPTLC-DPPH analyses of the compounds investigated and the STSs of flower pollen and bee pollen samples were performed to obtain additional information about the antioxidant activity of the separated compounds on the HPTLC plate. Such information could not be obtained for the STSs of the flower pollen and bee pollen samples by spectrophotometric assays. HPTLC-DPPH analyses and all spectrophotometric analyses performed in this study showed that phenolic acids have a much higher antioxidant activity than flavonoids. This observation is evident in Figure 4, where the highest intensity of the yellow-colored chromatographic zone was obtained for chlorogenic acid (Figure 4, track 2) and slightly lower intensity was obtained for 3,5-dicaffeoylquinic acid (Figure 4, track 4) at the same amount (0.4 µg) applied on the plate. The yellow zones of the STS of flower pollen (Figure 4, tracks 7 and 10) were ranked on intensity: the most intense zone was at R_F_ ≈ 0.58 for 3,5-dicaffeoylquinic acid (Figure 4, track 4), followed by the zone at the R_F_ ≈ 0.22 for chlorogenic acid (Figure 4, track 2), followed by other less intense zones at R_F_ ≈ 0.06 for quercetin-3-*O*-β-glucopyranosyl-(1→2)-β-galactopyranoside (Figure 4, track 1), R_F_ ≈ 0.52 for afzelin (Figure 4, track 3) and R_F_ ≈ 0.84 for platanoside (Figure 4, track 5). The highest intensity of the zone at R_F_ of 3,5-dicaffeoylquinic acid was expected as also the HPTLC-NP-PEG analyses of the STSs of the flower pollen (Figure 2, tracks 7 and 10) showed the most intense zones at the same R_F_ (light blue-colored zones).

The contribution of the antioxidant activity of the blue colored zones at R_F_ ≈ 0.25, 0.40, 0.45 and 0.65, other than chlorogenic acid and 3,5-dicaffeoylquinic acid, as seen in Figure 2 (tracks 7 and 10), was also detected by HPTLC-DPPH analysis. For the STSs of bee pollen samples from both countries, the most intense yellow zone was detected at R_F_ ≈ 0.06 for quercetin-3-*O*-β-glucopyranosyl-(1→2)-β-galactopyranoside. Related to the amounts of the compounds investigated, the STS of bee pollen from Slovenia (Figure 4, tracks 9 and 12) showed more intense zones than the STS of bee pollen from Türkiye (Figure 4, tracks 8 and 11), which was expected based on HPTLC-NP-PEG analyses (Figure 2, STS of bee pollen from Türkiye: tracks 8 and 11; STS of bee pollen from Slovenia: tracks 9 and 12).

**Figure 5:**
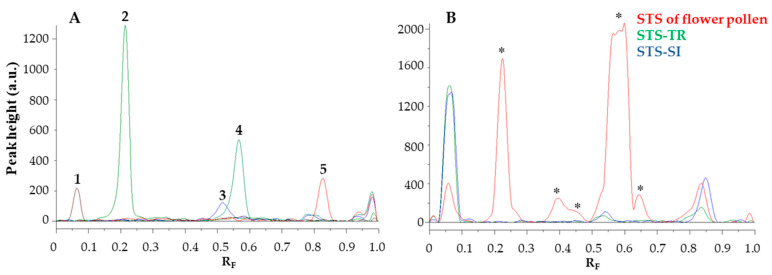
The videodensitograms were obtained in fluorescence mode by image analysis of the HPTLC silica gel F_254_ plate after HPTLC-DPPH analysis (Figure 4). (**A**): videodensitograms of investigated compounds quercetin-3-*O*-β-glucopyranosyl-(1→2)-β-galactopyranoside (peak 1: 0.4 µg), chlorogenic acid (peak 2: 0.4 µg), afzelin (peak 3: 0.4 µg), 3,5-dicaffeoylquinic acid (peak 4: 0.4 µg) and platanoside (peak 5: 0.4 µg). (**B**): STS of flower pollen (red line; 20 mg/mL, 5 µL), STS of bee pollen from Türkiye (green line; 20 mg/mL, 5 µL) and STS of bee pollen from Slovenia (blue line; 20 mg/mL, 5 µL). The asterisk (*) indicates the peaks that are specific for the flower pollen.

The image of the HPTLC plate (Figure 4) was converted to videodensitograms (Figure 5) in fluorescence mode. The videodensitograms of the STSs of bee pollen and flower pollen samples (Figure 5B) show peaks at R_F_ values of the compounds isolated (Figure 5A, R_F_ ≈ 0.06 for quercetin-3-*O*-β-glucopyranosyl-(1→2)-β-galactopyranoside, R_F_ ≈ 0.52 for afzelin and R_F_ ≈ 0.83 for platanoside). The videodensitograms of the STS from the flower pollen showed additional peaks at R_F_ values of chlorogenic acid (R_F_ ≈ 0.22) and 3,5-dicaffeoylquinic acid (R_F_ ≈ 0.58) (Figure 5B).

**Figure 6:**
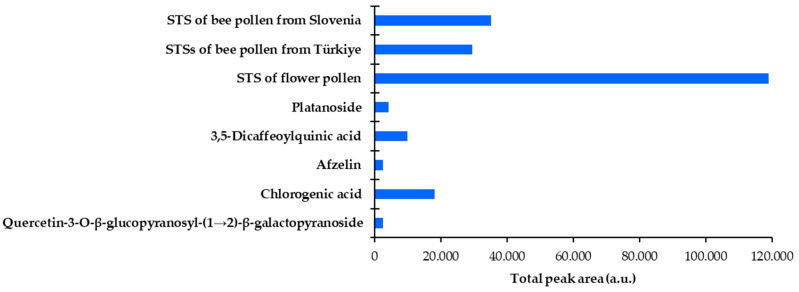
Total peak areas of all detected yellow bands in the videodensitograms of the compounds investigated (Figure 5A) and STSs of flower and bee pollen samples (Figure 5B). Videodensitograms were obtained by image analysis of the HPTLC silica gel F_254_ plate after HPTLC-DPPH analysis (Figure 4) with the following applications: quercetin-3-*O*-β-glucopyranosyl-(1→2)-β-galactopyranoside (0.4 µg) chlorogenic acid (0.4 µg) afzelin (0.4 µg), 3,5-dicaffeoylquinic acid (0.4 µg), platanoside (0.4 µg), STS of flower pollen (20 mg/mL, 5 µL), STS of bee pollen from Türkiye (20 mg/mL, 5 µL) and STS of bee pollen from Slovenia (20 mg/mL, 5 µL).

The videodensitograms (Figure 5) were used for the image analysis of the tracks of the compounds investigated and the tracks with the highest application of the STSs bee pollen (Figure 4, tracks 11 and 12) and flower pollen (Figure 4, track 10) samples. The total peak area obtained for the STS of flower pollen was significantly higher than the total peak areas obtained for the STSs of bee pollen (Figure 6). The lowest total peak area was obtained for the STS of bee pollen from Türkiye.

#### 3.5.2. HPTLC-XO Inhibitory Activity

**Figure 7:**
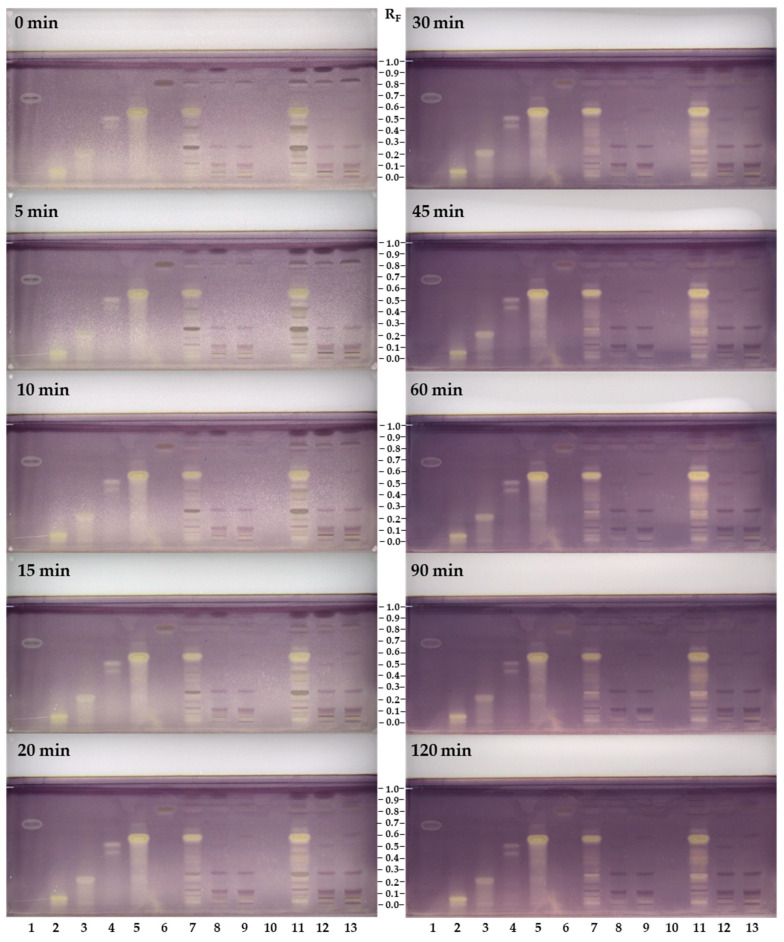
Influence of time on the detection of XO inhibitors on the HPTLC silica gel F_254_ plate developed with ethyl acetate–dichloromethane–acetic acid–formic acid–water (100:25:10:10:11, *v/v/v/v/v*) and documented at white light after HPTLC-XO assay at different time intervals (0–120 min). Applications: track 1: allopurinol (8 µg), track 2: quercetin-3-*O*-β-glucopyranosyl-(1→2)-β-galactopyranoside (8 µg); track 3: chlorogenic acid (8 µg); track 4: afzelin (8 µg); track 5: 3,5-dicaffeoylquinic acid (8 µg); track 6: platanoside (8 µg); tracks 7 and 11: sample test solution of the flower pollen (50 mg/mL, track 7 – 2 µL, track 11 – 5 µL); track 10: /; tracks 8 and 12: STS of the bee pollen from Türkiye (50 mg/mL, track 8 – 2 µL and track 12 – 5 µL); tracks 9 and 13: STS of the bee pollen from Slovenia (50 mg/mL, track 9 – 2 µL and track 13 – 5 µL).

Evaluation of the XO inhibitory activity was performed on HPTLC plates for all compounds investigated and sample test solutions (50 mg/mL) of flower pollen and bee pollen samples, which were all applied in higher concentrations than for HPTLC-NP-PEG and HPTLC-DPPH analyses. This could be because XO inhibitory activity is directly related to enzymatic activation. In HPTLC-XO inhibitory activity assay, the developed plate was dipped into a phosphate buffer solution containing xanthine and after incubation and drying was documented immediately (t = 0 min) and after 5, 10, 15, 20, 30, 45, 60, 90 and 120 min. Within this documentation interval, the plate was stored in the dark. The XO inhibitors were detected as white/yellow zones on a purple background (Figure 7). The positive control allopurinol appeared on the HPTLC plate immediately as a white zone, which lasted for 120 min (Figure 7, track 1).

Quercetin-3-*O*-β-glucopyranosyl-(1→2)-β-galactopyranoside (R_F_ ≈ 0.05, Figure 7, track 2), chlorogenic acid (R_F_ ≈ 0.21, Figure 7, track 3), afzelin (R_F_ ≈ 0.51, Figure 7, track 4) and 3,5-dicaffeoylquinic acid (R_F_ ≈ 0.57, Figure 7, track 5) appeared immediately as yellow zones and lasted for 120 min. Although platanoside (R_F_ ≈ 0.83, Figure 7, track 6) was applied on the plate in the same amount (8 µg) as the other compounds investigated, it showed the weakest XO inhibition activity, appearing as a yellow circle after 20 min.

Several yellow zones were detected in the sample test solution of flower pollen (Figure 7, tracks 7 and 11) and were ranked on intensity and the time of their detection: the most intense zone at R_F_ ≈ 0.57 of 3,5-dicaffeoylquinic acid (Figure 7, track 5) was detected at 0 min; this was followed by the zone of an unknown compound at R_F_ ≈ 0.25 at 90 min, which was not yellow earlier; followed by the zone at R_F_ ≈ 0.65 that appeared as a pale yellow at 15 min and its intensity increased after 30 min. The zones in-between R_F_ ≈ 0.3 and R_F_ ≈ 0.55 were not detected intensely until after 30 min (Figure 7, tracks 7 and 11). In the case of bee pollen samples (Figure 7, tracks 8, 9, 12 and 13), weak yellow zones appeared immediately (0 min) at R_F_ ≈ 0.05 for quercetin-3-*O*-β-glucopyranosyl-(1→2)-β-galactopyranoside (Figure 7, track 2). At the R_F_ value of platanoside (Figure 7, track 6) yellow zones appeared in all tracks of the sample test solutions (Figure 7, tracks 7–13). However, the occurrence of these yellow zones appearing at the same R_F_ value as platanoside could have resulted from other unknown compounds contributing to the activity.

It should be highlighted that enzymatic reactions are time-dependent, so documentation of the plate in certain time intervals is significant for the evaluation of the bioactive compounds. This is the first study emphasizing the importance of the time when the plate images in HPTLC-XO inhibitor analyses are captured. It should be noted that the time of capture of the plate images was not provided in the literature [35].

Spectrophotometric assays (Section 3.4.2. Xanthine Oxidase Inhibitory Activity and Superoxide Radicals Scavenging Activity) of the samples showed no XO inhibitory activity (Table 7). However, in HPTLC-XO analyses the contribution of both the extracts and the compounds in the extracts to the bioactivity was displayed. The reason for this difference is directly related to the sample concentration applied for analysis, which was much lower in the spectrophotometric analyses than in HPTLC-XO analyses. Testing samples in high concentration is the advantage of HPTLC-bioautography compared to spectrophotometric assays.

XO inhibitory activity of the compounds investigated obtained by HPTLC-XO was found to be comparable to the results of spectrophotometric tests (Table 7). Consequently, the compounds investigated appeared as intense yellow-colored zones on a purple background, except platanoside, which had a weak yellow zone.

## 4. Conclusions

In this study, bee pollen samples collected from Türkiye and Slovenia were evaluated. Palynological analysis was first used to identify their botanical source, which was ivy flower pollen. Then, the chemical profiles and pharmacological activities of the bee pollen samples, together with their botanical source, ivy flower pollen grains, were further investigated. HPTLC profiles of bee pollen samples revealed that samples of the same botanical origin exert similar chemical composition independent of where they were collected. Quercetin-3-*O*-β-glucopyranosyl-(1→2)-β-galactopyranoside, afzelin and platanoside were found as marker compounds for the identification of bee pollens from ivy flower. This study showed that HPTLC profiling could be an alternative to palynological analysis, and this approach can be applied to future studies of bee pollen. Among the marker compounds, quercetin-3-*O*-β-glucopyranosyl-(1→2)-β-galactopyranoside was found with the highest concentration in bee pollen samples, but not in the flower pollen. This difference may be the result of how honeybees produce bee pollen. It is interesting that quercetin-3-*O*-β-glucopyranosyl-(1→2)-β-galactopyranoside was the most bioactive one among marker compounds. It showed the highest antioxidant activity by *in vitro* DPPH, CUPRAC, FRAP and SOD activity tests. Additionally, it showed the highest XO inhibitory activity. HPTLC-bioautography (HPTLC-DPPH and HPTLC-XO) also confirmed its contribution to the bioactivity of the extract. To the best of our knowledge, this is the first report comparing chemical profiles and related bioactivities of the flower pollen and bee pollen of the same botanical origin, as well as the first report of the chemical profile and related bioactivities of ivy flower pollen. This study is important as the determination of the botanical sources of bee pollen should be taken into consideration for the standardization of bee pollen extracts. Therefore, more research on marker compounds in bee pollen, as well as on flower pollen and bee pollen of the same biological origin, is needed. The findings of this study can be applied to apitherapy and manufacturing of bee pollen-based food supplements.

## Figures and Tables

**Table 1:** Characterization of compounds isolated (Figure 1) using direct infusion HESI-MS^n^ in negative ion mode.

Compounds	MS (*m/z*) [Relative Intensity]	MS^n^ (*m/z*) [Relative Intensity]
Quercetin-3-*O*-β- glucopyrnosyl-(1→2)- β-galactopyranoside	625 [100], 626 [41], 627 [10], 723 [6], 661 [3], 1251 [3]	MS^2^ [625]: 300 [100], 301 [73], 445 [33], 505 [17], 271 [16], 463 [11], 355 [9], 255 [8], 343 [8], 299 [6], 325 [5], 273 [4], 409 [4], 427 [4], 367 [3]
		MS^3^ [625 → 300]: 271 [100], 255 [61], 272 [12], 254 [7], 243 [3]
		MS^2^ [723]: 625 [100], 399 [34], 437 [21], 687 [21], 685 [21]. 437 [21] MS^3^ [723 → 625]: 300 [100], 301 [76], 445 [40], 271 [17], 505 [17], 463 [13] MS^2^ [661]: 625 [100] MS^3^ [661 → 625]: 300 [100]
Platanoside	723 [100], 724 [49], 725 [13], 759 [13], 739 [9], 1447 [5]	MS^2^ [723]: 437 [100], 285 [36], 577 [4] MS^3^ [723 → 437]: 145 [100], 291 [72], 163 [71], 273 [63], 187 [23], 211 [16], 229 [14], 419 [14], 201 [10], 375 [7] MS^2^ [759]: 723 [100] MS^3^ [759 → 723]: 437 [100], 285 [31], 577 [4] MS^4^ [759 → 723 → 437]: 145 [100], 291 [70], 163 [68], 273 [54], 187 [21], 211 [16], 229 [15], 419 [14], 201 [8] MS^2^ [739]: 453 [100], 593 [24], 285 [19], 301 [14], 307 [4], 437 [3], 300 [2] MS^3^ [739 → 453]: 307 [100], 161 [45], 179 [34], 289 [22], 163 [13], 291 [12], 1445 [10], 217 [9], 263 [7], 135 [6], 227 [6] MS^4^ [739 → 453 → 307]: 161 [100], 135 [39], 179 [34]
Afzelin	431 [100], 432 [27], 863 [19], 467 [15], 630 [9], 285 [3]	MS^2^ [431]: 285 [100], 284 [44], 327 [5] MS^3^ [431→285]: 257 [100], 229 [46], 267 [40], 241 [30], 213 [20], 285 [18], 197 [18], 239 [15], 163 [15] MS^4^ [431→285 → 257]: 229 [100], 163 [67], 239 [38], 213 [22], 185 [11], 187 [9], 189 [9], 257 [7] MS^2^ [863]: 701 [100], 717 [97], 727 [64], 697 [32], 831 [24], 571 [17], 561 [17] 285 [6], 431 [2] MS^2^ [467]: 431 MS^3^ [467 → 431]: 285 [100], 327 [8], 255 [6]

**Table 2:** Linearity, LOD, and LOQ data for the compounds investigated.

Standards	Linearity Range (µg/mL)	r^2^	S *	Intercept	SD **	LOD (µg/mL)	LOQ (µg/mL)
Chlorogenic acid	0.5–50	0.9995	20.590	2.950	0.186	0.027	0.090
Quercetin-3-*O*-β-glucopyranosyl- (1→2)-β-galactopyranoside	0.5–50	0.9998	7.899	2.244	0.058	0.022	0.073
3,5-Dicaffeoylquinic acid	1–100	0.9994	31.248	9.637	0.790	0.076	0.253
Afzelin	1–50	0.9974	23.451	30.016	0.266	0.041	0.136
Platanoside	0.5–50	0.9998	38.132	0.982	0.271	0.021	0.071

* S: Slope; ** SD: Standard deviation of intercept.

**Table 3:** Intraday and interday precision data.

		Intraday Precision		Interday Precision	
Standards	Concentration (µg/mL)	Average Concentration (µg/mL ± SD) (*n* = 3)	RSD (%) (*n* = 3)	Average Concentration (µg/mL ± SD) (*n* = 3)	RSD (%) (*n* = 3)
Chlorogenic acid	5	5.283 ± 0.028	0.539	5.265 ± 0.014	0.266
		5.272 ± 0.008	0.160	5.269 ± 0.006	0.106
		5.259 ± 0.003	0.053	5.338 ± 0.023	0.430
Quercetin-3-*O*-β-glucopyraosyl- (1→2)-β-galactopyranoside	5	5.155 ± 0.032	0.618	5.172 ± 0.013	0.245
		5.142 ± 0.039	0.752	5.218 ± 0.015	0.280
		5.146 ± 0.013	0.246	5.311 ± 0.022	0.413
Platanoside	5	5.134 ± 0.007	0.135	5.165 ± 0.007	0.128
		5.141 ± 0.004	0.078	5.201 ± 0.008	0.162
		5.142 ± 0.007	0.135	5.263 ± 0.003	0.050
3,5-Dicaffeoylquinic acid	10	9.936 ± 0.033	0.335	9.921 ± 0.032	0.318
		9.907 ± 0.004	0.037	9.951 ± 0.020	0.201
		9.904 ± 0.002	0.019	10.052 ± 0.043	0.424
Afzelin	10	9.492 ± 0.020	0.206	9.526 ± 0.004	0.045
		9.489 ± 0.002	0.026	9.590 ± 0.011	0.118
		9.501 ± 0.004	0.045	9.704 ± 0.009	0.091

**Table 4:** Recovery of the compounds investigated.

	Added Concentration (µg/mL)	Recovery (%) (*n* = 3)			Added Concentration (µg/mL)	Recovery (%) (*n* = 3)	
Samples		Chlorogenic Acid	Quercetin-3-*O*-β- glucopyranosyl-(1→2)-β-galactopyranoside	Platanoside		3,5-Dicaffeoyl- quinic Acid	Afzelin
BP-TR	12.5	103.9	102.9	104.9	25	108.4	95.8
	6.25	97.3	104.4	94.6	12.5	90.9	82.6
	3.125	94.0	102.6	105.9	6.25	72.3	85.0
BP-SI	12.5	100.8	98.6	105.0	25	107.2	95.2
	6.25	103.6	102.5	107.4	12.5	91.8	87.1
	3.125	103.8	106.1	105.9	6.25	72.2	92.4
FP	12.5	100.6	96.3	93.1	25	83.8	78.4
	6.25	97.3	93.7	104.4	12.5	99.6	86.3
	3.125	90.3	80.0	96.6	6.25	76.9	87.7

**Table 5:** Contents of compounds investigated in bee pollen (BP-TR and BP-SI) and flower pollen (FP) samples.

Samples	Chlorogenic Acid	Quercetin-3-*O*-β- glucopyranosyl-(1→2)-β- galactopyranoside	Platanoside	3,5-Dicaffeoylquinic Acid	Afzelin
	mg/g ± SD (*n* = 3)				
BP-TR	N.d.	29.041 ± 0.088 ^a^	2.030 ± 0.009 ^c^	N.d.	2.052 ± 0.002 ^c^
BP-SI	N.d.	24.400 ± 0.211 ^b^	7.283 ± 0.107 ^a^	N.d.	2.419 ± 0.007 ^b^
FP	3.480 ± 0.010	3.959 ± 0.082 ^c^	6.242 ± 0.039 ^b^	22.372 ± 0.032	6.598 ± 0.010 ^a^

N.d.: Not detected. Different letters “a–c” in the same column indicate statistically significant differences (*p* ≤ 0.05).

**Table 6:** *In vitro* antioxidant activity of the compounds investigated and samples of ivy flower pollen (FP) and bee pollen (BP-TR and BP-SI) samples determined by DPPH, FRAP, ABTS and CUPRAC assays.

Compounds and Samples	DPPH	FRAP	ABTS	CUPRAC
	mg TE/g (*n* = 3)			
Quercetin-3-*O*-β-glucopyranosyl- (1→2)-β-galactopyranoside	400.21 ± 11.78 ^c^	227.30 ± 2.65 ^c^	242.13 ± 5.88 ^c^	560.53 ± 14.74 ^c^
Chlorogenic acid	812.72 ± 4.99 ^a^	676.51 ± 2.34 ^a^	803.52 ± 1.12 ^a^	950.43 ± 7.64 ^a^
Afzelin	149.35 ± 4.28 ^d^	58.54 ± 2.37 ^d^	104.71 ± 0.25 ^e^	171.40 ± 8.24 ^e^
3,5-Dicaffeoylquinic acid	665.53 ± 1.96 ^b^	542.36 ± 15.41 ^b^	701.64 ± 13.63 ^b^	760.83 ± 12.84 ^b^
Platanoside	143.11 ± 1.31 ^d^	49.06 ± 0.91 ^de^	173.15 ± 0.86 ^d^	213.68 ± 8.41 ^d^
FP	42.28 ± 0.68 ^e^	34.49 ± 0.05 ^e^	41.82 ± 2.87 ^f^	98.96 ± 4.22 ^f^
BP-TR	11.00 ± 0.04 ^f^	6.99 ± 0.12 ^f^	6.24 ± 0.14 ^g^	22.20 ± 1.31 ^g^
BP-SI	14.61 ± 0.75 ^f^	7.61 ± 0.27 ^f^	9.35 ± 0.15 ^g^	30.35 ± 1.56 ^g^

Different letters “a–g” in the same column indicate statistically significant differences (*p* ≤ 0.05).

**Table 7:** The results of XO inhibitory and SOD activities.

Compounds and DSTSs	XO Inhibitory Activity	SOD Activity
	IC_50_ (µg/mL ± SD) (*n* = 3)	
Quercetin-3-*O*-β-glucopyranosyl-(1→2)-β-galactopyranoside	73.01 ± 3.42 ^d^	5.09 ± 0.36 ^a^
Chlorogenic acid	12.68 ± 1.12 ^b^	1.58 ± 0.09 ^a^
Afzelin	14.46 ± 1.47 ^bc^	20.61 ± 1.30 ^b^
3,5-Dicaffeoylquinic acid	17.47 ± 2.23 ^c^	5.87 ± 0.32 ^a^
Platanoside	N.d.	N.d.
FP	N.d.	36.92 ± 3.49 ^c^
BP-TR	N.d.	63.08 ± 4.00 ^e^
BP-SI	N.d.	48.14 ± 2.42 ^d^
Allopurinol	2.25 ± 0.04 ^a^	3.03 ± 0.10 ^a^

N.d.: Not detected. Different letters “a–e” in the same column indicate statistically significant differences (*p* ≤ 0.05).

## Data Availability

The data presented in this study are available upon request from the corresponding author.
